# Association between segmental body composition and bone mineral density in US adults: results from the NHANES (2011–2018)

**DOI:** 10.1186/s12902-023-01506-z

**Published:** 2023-11-09

**Authors:** Yanze Lin, Xun Wang, Ruiji Wu, Jinlei Zhou, Fabo Feng

**Affiliations:** 1 Center for Plastic & Reconstructive Surgery, Department of Orthopedics, Zhejiang Provincial People’s Hospital (Affiliated People’s Hospital, Hangzhou Medical College), Hangzhou, Zhejiang China; 2 Second Clinical Medical College, Zhejiang Chinese Medical University, Hangzhou, Zhejiang China; 3 Orthopedic Department, Taizhou Hospital Affiliated to Wenzhou Medical University, Linhai, China

**Keywords:** Segmental body composition, Obesity, Bone mineral density, NHANES, Cross-sectional study

## Abstract

**Objective:**

The association between segmental body composition and bone mineral density (BMD) remains uncertain. The primary aim of this cross-sectional investigation was to elucidate the connection between segmental body composition and BMD within the United States adult population.

**Methods:**

We selected a cohort of 10,096 individuals from the National Health and Nutrition Examination Survey (NHANES) database, with a mean age of 39 years and a mean BMI of 28.5 kg/m². The parameter of segmental body composition was achieved by quantifying body fat and lean mass percentages across various anatomical regions, including the torso, Android, Gynoid, arms and legs. We conducted a weighted multivariate linear regression analysis to investigate the association between segmental body composition and total BMD. Additionally, subgroup analysis was performed based on age and gender.

**Results:**

We found an inverse association between fat proportion in each anatomical region and total BMD, with the arm and leg regions demonstrating the most significant negative correlation. Conversely, a positive correlation was observed between lean mass and BMD across all anatomical regions. These associations remained consistent in subgroup analyses.

**Conclusion:**

Our investigation revealed a negative association between adipose levels in various anatomical regions and BMD among Americans aged 20 to 59. Importantly, higher fat proportion in the extremities exerted the most deleterious impact on BMD. Furthermore, an increase in lean mass within each anatomical region was ascertained to confer a positive effect on bone health. Consequently, the evaluation of segmental body composition is well-positioned to predict bone health status.

## Introduction

Osteoporosis is a metabolic skeletal disorder characterized by a reduction in bone mass and deterioration of bone microstructure, leading to weakened bone strength and significantly heightened susceptibility to low-energy or fragile fractures [1]. Osteopenia and osteoporosis collectively afflict 53.4 million elderly individuals in the United States, with their prevalence expected to rise as the population ages [2]. Osteoporosis-induced fractures in the United States occur approximately 1.5 million times each year [3]. The economic burden of treating fractures arising from osteoporosis is projected to reach nearly 50 billion by 2040, imposing a substantial strain on the American economy and society [4]. A decrease in bone mineral density (BMD), a reliable indicator of osteoporosis, is intrinsically associated with an elevated fracture risk [5, 6]. Consequently, the identification of risk factors correlated with BMD decline assumes paramount importance in the prediction and prevention of osteoporosis.

Obesity is a chronic metabolic condition influenced by an interplay of environmental and genetic variables, characterized by an abnormal or excessive accumulation of adipose tissue [7]. Over the last three decades, the prevalence of overweight and obesity in the United States has surged to alarming proportions. This widespread epidemic has given rise to a multitude of comorbidities, including an elevated susceptibility to metabolic disorders, cardiovascular disease, and mortality [8]. Despite an expanding body of evidence that highlights the connection between obesity and various health outcomes, a considerable debate lingers concerning the association between obesity and bone health [9]. Traditionally, it was believed that obesity provided protection against osteoporosis [10, 11], but an increasing body of research has demonstrated that adipose tissue does not confer benefits to bone health [12]. Body mass index (BMI) is commonly utilized to gauge obesity status, but its inability to accurately reflect body composition and predict metabolic disease risk has been questioned [13, 14]. Therefore, numerous studies have proposed that the assessment of body composition, a cornerstone of human metabolism and physiology [15], holds the potential to significantly enhance our understanding of obesity, metabolic health, chronic diseases, aging, and the intricate relationships between metabolic disorders and fat distribution [16–18].

The synthesis and metabolism of adipose cytokines exhibit a remarkable degree of variability depending on the specific location of the adipose tissue [19]. Consequently, a more effective approach for assessing and predicting metabolic disorders involves the meticulous examination of segmental body composition [20, 21]. Recent research has illuminated distinct relationships between the accumulation of adipose tissue in the upper and lower body about the risk of obesity-related metabolic disorders and comorbidities [22]. However, a lack of comprehensive studies centered on segmental body composition and its connection to BMD has left the association between segmental body composition and BMD shrouded in uncertainty. In a cross-sectional study conducted in southern Sri Lanka, a significant positive correlation emerged between lean and fat mass in different body areas and BMD among adults aged 30–54 years [23]. However, a prospective community-based cohort investigation explored the relationship between bone strength and fat mass in various areas of the body regions, revealing a negative correlation with central fat, such as Android fat, while simultaneously demonstrating a positive correlation with fat mass in the leg and Gynoid region [24]. Therefore, our endeavor aims to assess the proportions of fat and lean mass in various body regions to foster a deeper understanding of the association between segmental body composition and BMD.

## Materials and methods

### Study population

The National Health and Nutrition Examination Survey (NHANES) is a sophisticated and comprehensive research effort known for its multifaceted, multi-stage probabilistic sampling design, which enables the collection of diverse samples representing the U.S. population accurately. By conducting in-person interviews and standardized physical examinations within cutting-edge mobile screening facilities, NHANES surpasses the limitations of conventional survey methodologies, resulting in a rich database covering nutrition, health, and a myriad of other parameters. This invaluable dataset supports the estimation of disease prevalence and incidence rates, making NHANES a potent tool for policymakers in developing well-informed and effective public health policies for the broader population [25, 26].

**Fig. 1:**
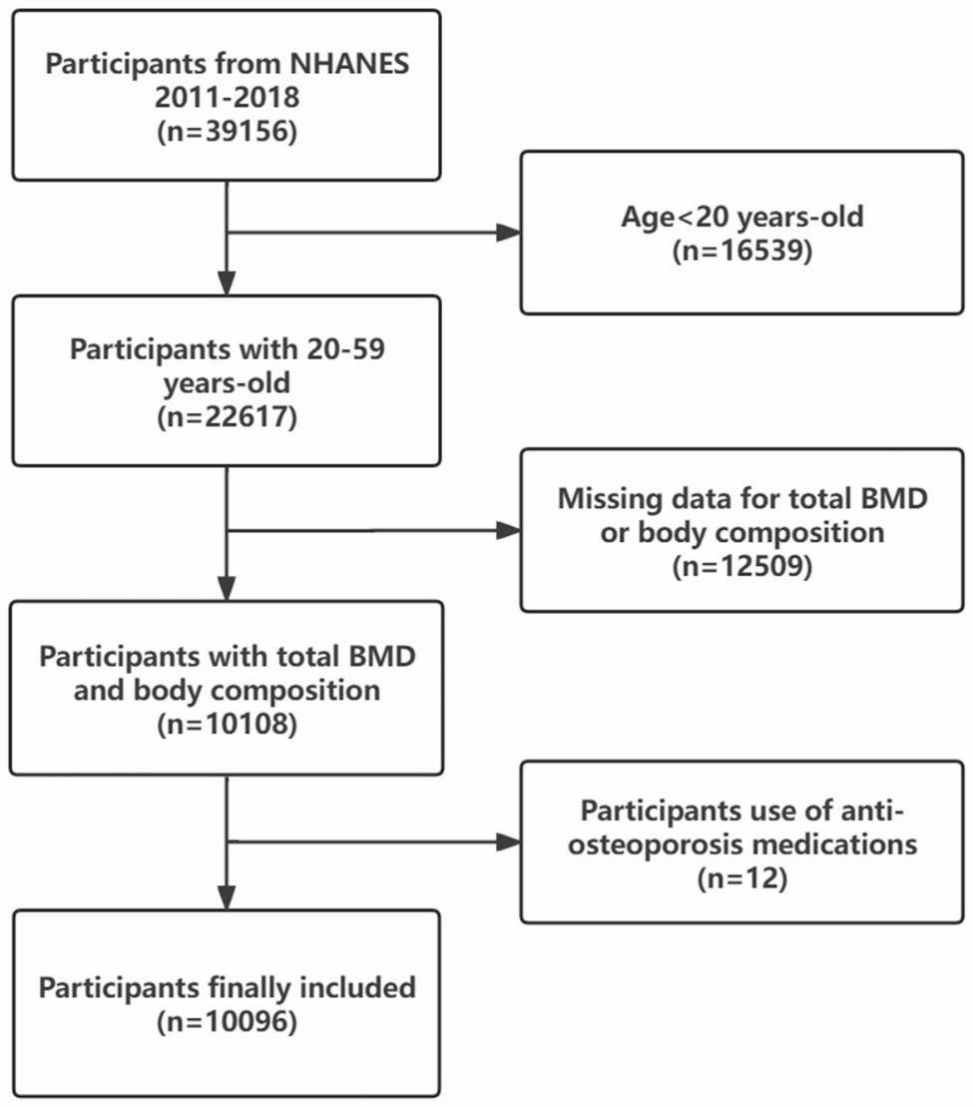
Flowchart of study participants

Dual-energy X-ray Absorptiometry (DXA) represents a medical imaging technique typically restricted to individuals aged 8 to 59 years for various reasons. In our investigation, we focused our attention exclusively on individuals aged 20 to 59 years who met the criteria for undergoing DXA examinations from 2011 to 2018. Specifically, pregnant women, individuals exceeding 450 pounds in weight, those taller than 6 feet 5 inches, and individuals who had recently undergone radiation contrast agent (barium) treatments were ineligible for DXA examinations. Furthermore, certain DXA results were considered invalid due to issues like excessive X-ray noise, problems with positioning resulting from excessive scanning areas, overlapping limbs, and pathological obesity. Participants without data on total BMD, segmental body composition, or those taking anti-osteoporosis medications were systematically excluded. As a result, the final cohort comprised 10,096 participants for the study. (Fig. 1)

### Exposure-segmental body composition

DXA examination was utilized to assess the segmental body compositions conducted by a team of rigorously trained and certified radiology technologists using Hologic QDR-4500 A fan-beam densitometers (Hologic; Bedford, MA, USA). All DXA examination data were meticulously analyzed with Hologic APEX software(version 4.0), which was proficient in quantifying multiple regional components, including fat and lean soft tissue. Fat and lean mass were measured in various anatomical regions, encompassing the torso, Android, Gynoid, legs, and arms.

**Fig. 2:**
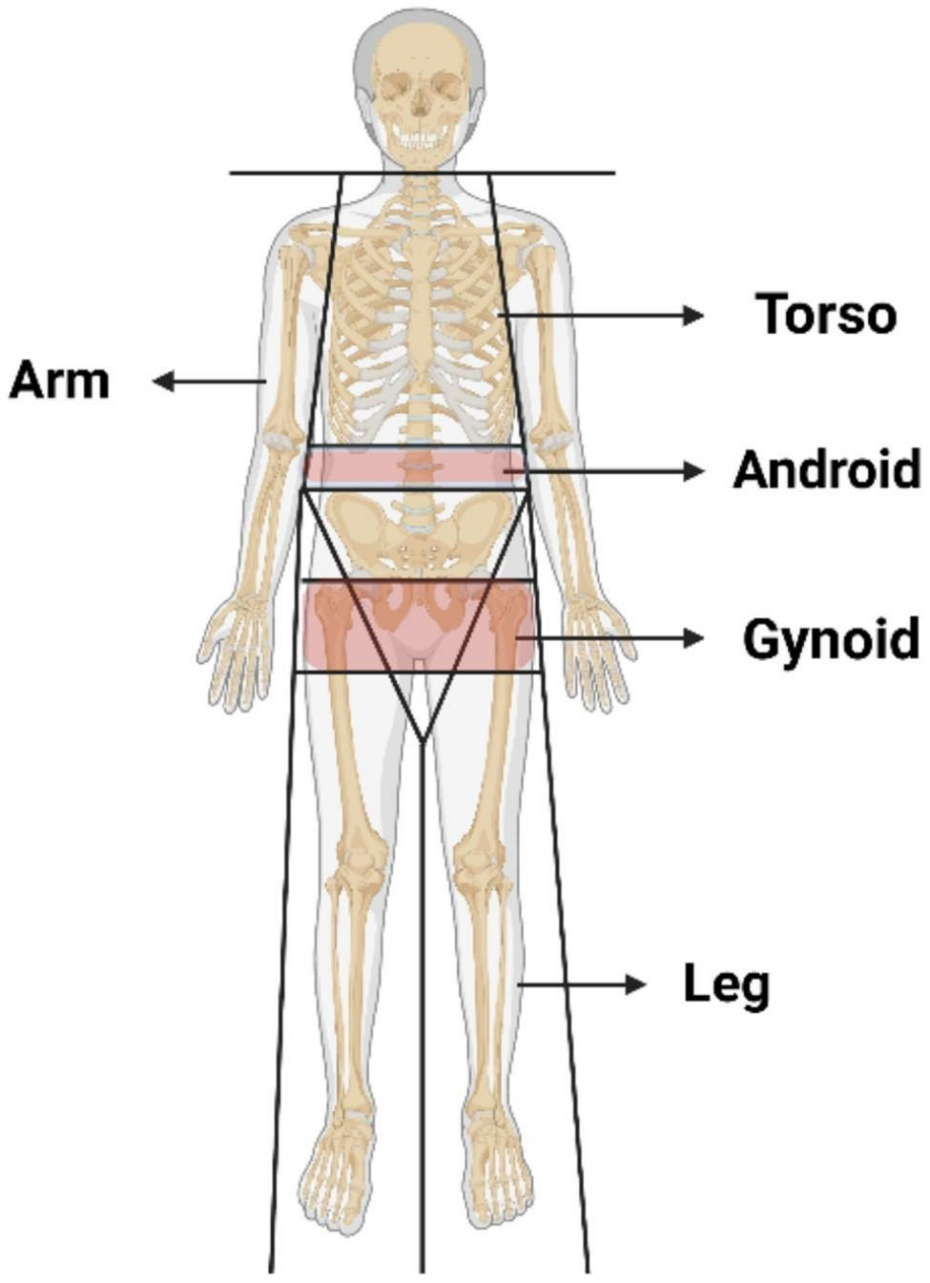
Body region distribution

The torso area is delineated as the area from the lower edge of the chin to the lower perimeter of the diagonal line extending through the femoral neck and converging below the pubic symphysis, with a vertical boundary outside the ribs. The area below the lower edge of the torso is designated as the leg area [27]. The Android area is the lower torso area surrounded by two lines: the horizontal line positioned beneath the pelvis and the line automatically situated above the pelvic line. The Gynoid area is defined by the upper and lower lines. The upper line measures 1.5 times the height of the Android area below the pelvic line, and the lower line measures twice the height of the Android area [28] (Fig. 2). The fat mass percentage (FM%) and lean mass percentage (LM%) are calculated by dividing the respective fat or lean mass by the total mass of the corresponding body segment weight. For instance, Torso FM% is derived by dividing the fat mass within the torso by the total mass of the torso.

### Outcome-BMD

The participants’ BMD was measured using the same DXA technology employed for evaluating segmental body compositions. DXA scans provided comprehensive bone assessments, including data on total BMD. For further details on inspection protocols and quality control, please refer to the NHANES website(www.cdc.gov/nchs/nhanes/index.htm).

### Covariates

We collected demographic variables, including age, gender (male and female), race(Mexican American, other Hispanic, Non-Hispanic White, Non-Hispanic Black, other race), education level(less than high school, high school, more than high school), and family income to poverty ratio. Furthermore, comprehensive physical assessments were conducted, which included the measurements of weight(kg), height(cm), body mass index(BMI)(kg/m^2^), arm circumference(cm), and waist circumference(cm). The BMI was calculated through the division of an individual’s weight(kg) by the square of their height(m^2^). In addition, participants were categorized into three distinct groups predicated upon smoking status: never smoked, formerly smoked, and current smoking. Alcohol consumption was quantified in terms of the frequency of alcohol use over a year. Physical activity levels were stratified by Physical Activity Guidelines, with recommendations of ≥ 75 min per week of vigorous or ≥ 150 min per week of moderate physical activity: active (meeting or exceeding the level of recommended activity), less active (below the recommended activity level), and inactive (no engagement in physical activity) [29]. Moreover, dietary intake of protein, calcium, and phosphorus was assessed through two separate 24-hour food recall interviews. Hypertension was defined as individuals with an average systolic or diastolic blood pressure exceeding 140/90 mm/Hg, as measured on three separate occasions, or those prescribed antihypertensive medications. Participants with glycosylated hemoglobin (HbA1c) levels ≥ 6.5%, and those under medication for diabetes management were classified as having diabetes.

### Statistical analysis

The data underwent rigorous processing following the stipulated protocols of the NHANES database. All subsequent analyses incorporated the sample weights and multi-period combination weights. Continuous variables were presented as mean values with corresponding standard deviations, and P-values were calculated using a weighted linear regression model. Categorical variables were expressed as percentages, and the corresponding P-values were calculated via the weighted chi-square test.

**Fig. 3:**
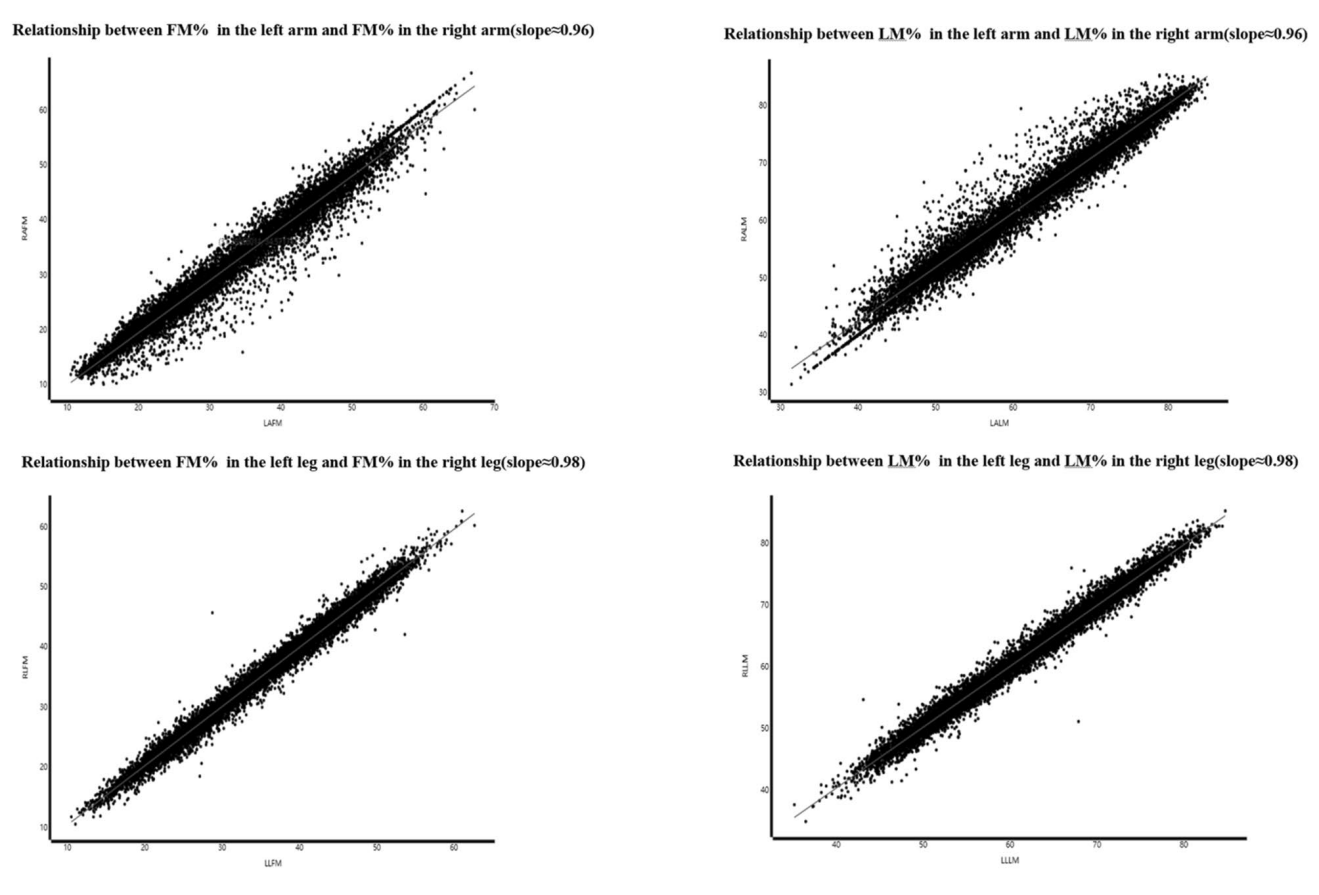
Relationship of FM% and LM% in the left and right

Our preliminary analysis revealed a strong correlation between the FM% and LM% in both the left and right arms and legs (Fig. 3). Therefore, the FM% and LM% values for the overall arms and legs were represented by averaging the values from both the left and the right sides. Given the non-normal distribution of data concerning body composition measurements, FM% and LM% values for the arms, legs, Android area, Gynoid region, and torso, as well as the total FM% and LM%, were expressed in quartiles for this study. The first quartile served as a reference point to elucidate the relationship between body composition and BMD.

A weighted multiple linear regression model was employed to evaluate the association between segmental body composition quartiles and total BMD. The results were presented as β, 95% confidence interval (CI), and P-values. In Model 1, no covariate adjustments were made to comprehensively examine the association. Model 2 incorporated adjustments for age and gender to account for potential confounding effects. Building upon Model 2, Model 3 introduced additional adjustments that included variables such as race, education level, family income to poverty ratio, smoking status, alcohol consumption, physical activity, protein intake, calcium intake, phosphorus intake, hypertension, diabetes, height, weight, BMI, arm circumference, and waist circumference.

Subsequent analyses were stratified based on age and gender. All statistical analyses were conducted utilizing EmPower Stats (https://www.empowerstats.com) and R software (version 3.6.3). A p-value less than 0.05 was considered statistically significant.

## Results

### Characteristics of the selected participants

The characteristics of the subjects, stratified by quartiles of total BMD (Q1:0.697-1.039 g/ cm²; Q2:1.039–1.108 g/ cm²; Q3: 1.108-1.18 g/ cm²; Q4: 1.18-1.88 g/ cm²), were summarized in Table 1. The study cohort comprised a total of 10,096 participants with an average age of 39 years and an average BMI of 28.5 kg/m². Notably, discernible disparities were observed among the BMD quartile groups in terms of personal habits, demographic characteristics, comorbidities, and physical measurements, with the exception of smoking status. Participants in the lowest BMD quartile were more likely to be older Caucasian women of shorter stature, lower body weight, reduced BMI, diminished arm and waist circumference, and had achieved a lower level of educational attainment. Additionally, these participants demonstrated lower daily consumption of alcohol, protein, calcium, and phosphorus, coupled with higher rates of smoking and physical inactivity.

**Table 1:** The characteristics of participants included in this study (n = 10,096),NHANES 2011–2018

Total BMD(g/cm²)	Q1(0.697–1.039)	Q2(1.039–1.108)	Q3(1.108–1.18)	Q4(1.18–1.88)	P value
N, unweighted	2504	2525	2532	2535	
Demographic data					
Age(years)	41.45 ± 12.49	38.17 ± 11.69	38.28 ± 11.10	38.75 ± 11.24	< 0.001
Gender(%)					< 0.001
Male	31.66	43.12	56.73	72.78	
Female	68.34	56.88	43.27	27.22	
Race(%)					< 0.001
Mexican American	12.37	10.69	11.66	7.88	
Other Hispanic	9.01	8.08	7.13	5.44	
Non-Hispanic White	60.57	62.74	61.71	58.95	
Non-Hispanic Black	5.42	7.84	10.96	20.08	
Other Race	12.63	10.65	8.54	7.65	
Family income-to-poverty ratio	2.82 ± 1.63	2.90 ± 1.63	2.92 ± 1.60	3.04 ± 1.61	< 0.001
Education level(%)					< 0.001
Lower than high school	15.57	12.93	13.41	11.14	
High school	21.79	20.08	23.15	20.34	
More than high school	62.64	66.99	63.44	68.52	
Personal habits					
Smoke(%)					0.077
Never	57.57	61.82	57.66	59.59	
Ever	19.87	17.91	20	20.11	
Current	22.56	20.27	22.34	20.3	
Alcohol use days per year	2.76 ± 1.95	2.86 ± 2.37	3.03 ± 2.24	3.06 ± 2.08	< 0.001
Physical activity(%)					< 0.001
Inactive	56.94	53.03	51.04	45.99	
Less active	24.32	22.54	23.05	21.98	
Active	18.74	24.43	25.91	32.03	
Protein intake(g)	77.59 ± 28.77	82.67 ± 31.93	86.00 ± 33.49	92.56 ± 35.51	< 0.001
Calcium intake(mg)	903.37 ± 395.63	967.19 ± 468.14	994.65 ± 478.59	1065.91 ± 495.29	< 0.001
Phosphorus intake(mg)	1303.87 ± 458.41	1386.45 ± 531.55	1436.70 ± 552.73	1543.45 ± 581.23	< 0.001
Comorbidities					
Hypertension(%)					< 0.001
Yes	14.46	11.75	11.73	15.45	
No	85.54	88.25	88.27	84.55	
Diabetes(%)					< 0.001
Yes	5.63	6.09	5.87	7.71	
No	94.37	93.91	94.13	92.29	
Body examination data					
BMI (kg/m^2^)	27.20 ± 6.33	28.58 ± 6.74	28.62 ± 6.42	29.66 ± 6.22	< 0.001
Weight(kg)	73.49 ± 18.33	80.21 ± 19.81	82.97 ± 19.58	89.64 ± 19.75	< 0.001
Height(cm)	164.29 ± 8.89	167.50 ± 8.74	170.26 ± 8.98	173.83 ± 8.40	< 0.001
Arm circumference(cm)	31.38 ± 4.68	32.91 ± 4.86	33.45 ± 4.69	35.05 ± 4.59	< 0.001
Waist circumference(cm)	93.97 ± 15.50	96.77 ± 16.05	97.43 ± 15.71	99.64 ± 15.30	< 0.001
Android fat mass(%)	36.58 ± 8.68	35.83 ± 8.95	34.35 ± 8.95	32.44 ± 8.71	< 0.001
Android lean mass(%)	63.42 ± 8.68	64.17 ± 8.95	65.65 ± 8.95	67.56 ± 8.71	< 0.001
Gynoid fat mass(%)	38.48 ± 8.02	36.59 ± 8.52	34.49 ± 8.36	31.45 ± 8.10	< 0.001
Gynoid lean mass(%)	61.52 ± 8.02	63.41 ± 8.52	65.51 ± 8.36	68.55 ± 8.10	< 0.001
Arm fat mass(%)	37.40 ± 10.67	35.28 ± 11.21	32.30 ± 10.94	28.89 ± 10.45	< 0.001
Arm lean mass(%)	59.05 ± 10.28	61.01 ± 10.82	63.78 ± 10.55	66.99 ± 10.05	< 0.001
Leg fat mass(%)	38.77 ± 9.11	36.56 ± 9.63	34.02 ± 9.36	30.62 ± 9.01	< 0.001
Leg lean mass(%)	58.26 ± 8.78	60.26 ± 9.22	62.56 ± 8.92	65.66 ± 8.53	< 0.001
Torso fat mass(%)	33.86 ± 8.21	32.80 ± 8.45	31.17 ± 8.42	29.02 ± 8.28	< 0.001
Torso lean mass(%)	64.75 ± 7.99	65.69 ± 8.23	67.22 ± 8.18	69.22 ± 8.00	< 0.001
Total fat mass(%)	35.47 ± 7.80	33.98 ± 8.26	31.95 ± 8.16	29.34 ± 7.97	< 0.001
Total lean mass(%)	61.86 ± 7.50	63.16 ± 7.95	65.00 ± 7.82	67.39 ± 7.60	< 0.001

Mean +/− SD for continuous variables, Percentage (%) for Categorical variables; P value was estimated using X² for proportions, T test for means; BMD, bone mineral density; BMI, body mass index (calculated as weight in kilograms divided by height in meters squared)

### The association between body composition parameters and total BMD

The association between body composition parameters and BMD was detailed in Table 2. In the unadjusted model, it was observed that the FM% of each body segment (Arm, Leg, Android region, Gynoid region, Torso) exhibited a negative correlation with BMD, while the LM% of each body segment showed a positive correlation with BMD. Importantly, even after adjusting the confounding variables in Model 2 and Model 3, this relationship remained robust. Furthermore, in Model 3, it was found that the highest quartile of Arm FM% [-0.0882,(-0.0983,-0.0780)] and leg FM% in the highest quartile [-0.0858,(− 0.0945,-0.0771)] had a more significant impact on BMD compared to other body regions.

**Table 2:** Association of body composition parameters with total BMD aged 20–59 years (n = 10,096),NHANES 2011–2018

Body composition parameters	Model 1 β (95% CI) P value	P trend	Model2 β (95% CI) P value	P trend	Model 3 β (95% CI) P value	P trend
Android FM%						
Q1(11.35–28.88)	reference	< 0.001	reference	< 0.001	reference	< 0.001
Q2(28.88–35.70)	-0.0124 (-0.0181, -0.0066) 0.000022		-0.0045 (-0.0100, 0.0011) 0.114710		-0.0153 (-0.0208, -0.0098) < 0.000001	
Q3(35.70–41.70)	-0.0254 (-0.0312, -0.0197) < 0.000001		-0.0074 (-0.0131, -0.0018) 0.010318		-0.0323 (-0.0388, -0.0258) < 0.000001	
Q4(41.70-59.15)	-0.0525 (-0.0583, -0.0468) < 0.000001		-0.0159 (-0.0219, -0.0098) < 0.000001		-0.0678 (-0.0762, -0.0593) < 0.000001	
Android LM%						
Q1(40.85–58.30)	reference	< 0.001	reference	< 0.001	reference	< 0.001
Q2(58.30–64.30)	0.0271 (0.0213, 0.0330) < 0.000001		0.0084 (0.0027, 0.0141) 0.003834		0.0355 (0.0296, 0.0413) < 0.000001	
Q3(64.30-71.12)	0.0402 (0.0344, 0.0460) < 0.000001		0.0114 (0.0056, 0.0172) 0.000133		0.0524 (0.0455, 0.0593) < 0.000001	
Q4(71.12–88.64)	0.0525 (0.0468, 0.0583) < 0.000001		0.0159 (0.0098, 0.0219) < 0.000001		0.0678 (0.0593, 0.0762) < 0.000001	
Gynoid FM%						
Q1(11.82–28.08)	reference	< 0.001	reference	< 0.001	reference	< 0.001
Q2(28.08–35.07)	-0.0210 (-0.0266, -0.0155) < 0.000001		-0.0129 (-0.0185, -0.0073) 0.000006		-0.0196 (-0.0251, -0.0142) < 0.000001	
Q3(35.07–42.53)	-0.0628 (-0.0685, -0.0572) < 0.000001		-0.0271 (-0.0346, -0.0196) < 0.000001		-0.0440 (-0.0516, -0.0364) < 0.000001	
Q4(42.53–61.90)	-0.0847 (-0.0903, -0.0790) < 0.000001		-0.0377 (-0.0461, -0.0292) < 0.000001		-0.0718 (-0.0808, -0.0629) < 0.000001	
Gynoid LM%						
Q1(38.10-57.47)	reference	< 0.001	reference	< 0.001	reference	< 0.001
Q2(57.47–64.93)	0.0218 (0.0162, 0.0274) < 0.000001		0.0106 (0.0049, 0.0163) 0.000260		0.0278 (0.0223, 0.0333) < 0.000001	
Q3(64.93–71.92)	0.0637 (0.0581, 0.0692) < 0.000001		0.0248 (0.0172, 0.0324) < 0.000001		0.0522 (0.0447, 0.0597) < 0.000001	
Q4(71.92–88.18)	0.0847 (0.0790, 0.0903) < 0.000001		0.0377 (0.0292, 0.0461) < 0.000001		0.0718 (0.0629, 0.0808) < 0.000001	
Arm FM%						
Q1(11.20-24.09)	reference	< 0.001	reference	< 0.001	reference	< 0.001
Q2(24.09–32.19)	-0.0209 (-0.0265, -0.0153) < 0.000001		-0.0105 (-0.0161, -0.0049) 0.000237		-0.0230 (-0.0287, -0.0173) < 0.000001	
Q3(32.19–42.79)	-0.0651 (-0.0707, -0.0595) < 0.000001		-0.0247 (-0.0319, -0.0175) < 0.000001		-0.0554 (-0.0634, -0.0475) < 0.000001	
Q4(42.79–66.77)	-0.0793 (-0.0850, -0.0736) < 0.000001		-0.0265 (-0.0347, -0.0183) < 0.000001		-0.0882 (-0.0983, -0.0780) < 0.000001	
Arm LM%						
Q1(31.44–53.66)	reference	< 0.001	reference	< 0.001	reference	< 0.001
Q2(53.66–63.92)	0.0125 (0.0069, 0.0182) 0.000013		-0.0011 (-0.0068, 0.0046) 0.703474		0.0238 (0.0178, 0.0298) < 0.000001	
Q3(63.92–71.85)	0.0564 (0.0508, 0.0620) < 0.000001		0.0089 (0.0013, 0.0165) 0.020958		0.0501 (0.0418, 0.0584) < 0.000001	
Q4(71.85–84.68)	0.0765 (0.0708, 0.0822) < 0.000001		0.0180 (0.0096, 0.0264) 0.000027		0.0674 (0.0572, 0.0775) < 0.000001	
Leg FM%						
Q1(10.80-26.68)	reference	< 0.001	reference	< 0.001	reference	< 0.001
Q2(26.68–34.47)	-0.0242 (-0.0298, -0.0186) < 0.000001		-0.0161 (-0.0217, -0.0104) < 0.000001		-0.0254 (-0.0308, -0.0199) < 0.000001	
Q3(34.47–42.94)	-0.0613 (-0.0669, -0.0557) < 0.000001		-0.0280 (-0.0352, -0.0208) < 0.000001		-0.0531 (-0.0604, -0.0457) < 0.000001	
Q4(42.94–61.84)	-0.0867 (-0.0923, -0.0811) < 0.000001		-0.0409 (-0.0491, -0.0327) < 0.000001		-0.0858 (-0.0945, -0.0771) < 0.000001	
Leg LM%						
Q1(35.74–54.11)	reference	< 0.001	reference	< 0.001	reference	< 0.001
Q2(54.11–62.16)	0.0242 (0.0187, 0.0298) < 0.000001		0.0109 (0.0052, 0.0166) 0.000193		0.0274 (0.0219, 0.0328) < 0.000001	
Q3(62.16–69.65)	0.0646 (0.0591, 0.0702) < 0.000001		0.0223 (0.0148, 0.0298) < 0.000001		0.0525 (0.0451, 0.0600) < 0.000001	
Q4(69.65–85.04)	0.0806 (0.0750, 0.0862) < 0.000001		0.0293 (0.0210, 0.0377) < 0.000001		0.0652 (0.0565, 0.0740) < 0.000001	
Torso FM%						
Q1(10.60-25.77)	reference	< 0.001	reference	< 0.001	reference	< 0.001
Q2(25.77–32.07)	-0.0200 (-0.0257, -0.0144) < 0.000001		-0.0114 (-0.0170, -0.0059) 0.000053		-0.0248 (-0.0304, -0.0193) < 0.000001	
Q3(32.07–38.58)	-0.0368 (-0.0425, -0.0311) < 0.000001		-0.0123 (-0.0181, -0.0065) 0.000032		-0.0416 (-0.0483, -0.0349) < 0.000001	
Q4(38.58–56.27)	-0.0626 (-0.0684, -0.0568) < 0.000001		-0.0152 (-0.0217, -0.0087) 0.000004		-0.0779 (-0.0870, -0.0687) < 0.000001	
Torso LM%						
Q1(42.78–60.06)	reference		reference		reference	< 0.001
Q2(60.06–66.40)	0.0248 (0.0190, 0.0307) < 0.000001		0.0015 (-0.0044, 0.0073) 0.623821		0.0312 (0.0250, 0.0375) < 0.000001	
Q3(66.40-72.55)	0.0431 (0.0373, 0.0488) < 0.000001		0.0028 (-0.0034, 0.0090) 0.370732		0.0458 (0.0383, 0.0532) < 0.000001	
Q4(72.55–86.90)	0.0594 (0.0536, 0.0652) < 0.000001		0.0097 (0.0032, 0.0163) 0.003548		0.0641 (0.0550, 0.0733) < 0.000001	
Total FM%						
Q1(11.69–26.29)	reference	< 0.001	reference	< 0.001	reference	< 0.001
Q2(26.29–32.33)	-0.0226 (-0.0282, -0.0170) < 0.000001		-0.0104 (-0.0160, -0.0048) 0.000264		-0.0251 (-0.0307, -0.0195) < 0.000001	
Q3(32.33–39.53)	-0.0595 (-0.0651, -0.0539) < 0.000001		-0.0243 (-0.0307, -0.0179) < 0.000001		-0.0593 (-0.0665, -0.0521) < 0.000001	
Q4(39.53–55.37)	-0.0770 (-0.0826, -0.0713) < 0.000001		-0.0248 (-0.0322, -0.0174) < 0.000001		-0.0943 (-0.1038, -0.0848) < 0.000001	
Total LM%						
Q1(42.99–57.75)	reference	< 0.001	reference	< 0.001	reference	< 0.001
Q2(57.75–64.70)	0.0159 (0.0102, 0.0216) < 0.000001		-0.0020 (-0.0078, 0.0038) 0.494247		0.0275 (0.0214, 0.0336) < 0.000001	
Q3(64.70–70.50)	0.0533 (0.0476, 0.0589) < 0.000001		0.0096 (0.0028, 0.0164) 0.005848		0.0553 (0.0476, 0.0631) < 0.000001	
Q4(70.50-84.37)	0.0730 (0.0673, 0.0787) < 0.000001		0.0157 (0.0082, 0.0233) 0.000047		0.0727 (0.0631, 0.0822) < 0.000001	

LM%, lean mass percentage; FM%, fat mass percentage

Model 1: No covariates were adjusted

Model 2: Adjusted for age and gender

Model 3: Adjusted for age, gender, race, poverty ratio, education level, smoke, alcohol, physical activity, Protein intake, Calcium intake, Phosphorus intake, Hypertension, Diabetes, BMI

Weight, Height, Arm circumference, Waist circumference

### The association between segmental body composition and total BMD by age and gender

We categorized our study participants into two age groups: 20–39 years old and 40–59 years old, and further stratified them by gender to conduct a subgroup analysis based on Model 3(Table 3). Our findings discovered the correlation between segmental body composition and BMD remained consistent across gender and age groups.

**Table 3:** Association of segmental body composition with total BMD, stratified by gender and age(n = 10,096),NHANES 2011–2018

	Male, β (95% CI) P value	Female, β (95% CI) P value	20–39 years, β (95% CI) P value	40–59 years, β (95% CI) P value
Android FM%				
Q1(11.35–28.88)	reference	reference	reference	reference
Q2(28.88–35.70)	-0.0196 (-0.0270, -0.0122) < 0.000001	-0.0071 (-0.0157, 0.0016) 0.109539	-0.0192 (-0.0263, -0.0122) < 0.000001	-0.0155 (-0.0242, -0.0068) 0.000468
Q3(35.70–41.70)	-0.0411 (-0.0507, -0.0315) < 0.000001	-0.0163 (-0.0254, -0.0072) 0.000446	-0.0327 (-0.0412, -0.0241) < 0.000001	-0.0357 (-0.0456, -0.0258) < 0.000001
Q4(41.70-59.15)	-0.0947 (-0.1089, -0.0805) < 0.000001	-0.0459 (-0.0567, -0.0351) < 0.000001	-0.0716 (-0.0831, -0.0601) < 0.000001	-0.0659 (-0.0784, -0.0535) < 0.000001
Android LM%				
Q1(40.85–58.30)	reference	reference	reference	reference
Q2(58.30–64.30)	0.0535 (0.0430, 0.0641) < 0.000001	0.0296 (0.0225, 0.0367) < 0.000001	0.0390 (0.0308, 0.0472) < 0.000001	0.0303 (0.0219, 0.0387) < 0.000001
Q3(64.30-71.12)	0.0751 (0.0633, 0.0869) < 0.000001	0.0389 (0.0298, 0.0479) < 0.000001	0.0524 (0.0428, 0.0619) < 0.000001	0.0505 (0.0405, 0.0604) < 0.000001
Q4(71.12–88.64)	0.0947 (0.0805, 0.1089) < 0.000001	0.0459 (0.0351, 0.0567) < 0.000001	0.0716 (0.0601, 0.0831) < 0.000001	0.0659 (0.0535, 0.0784) < 0.000001
Gynoid FM%				
Q1(11.82–28.08)	reference	reference	reference	reference
Q2(28.08–35.07)	-0.0160 (-0.0220, -0.0099) < 0.000001	0.0155 (-0.0164, 0.0473) 0.341850	-0.0187 (-0.0260, -0.0114) < 0.000001	-0.0187 (-0.0268, -0.0105) 0.000007
Q3(35.07–42.53)	-0.0412 (-0.0513, -0.0312) < 0.000001	-0.0038 (-0.0350, 0.0274) 0.810883	-0.0411 (-0.0510, -0.0312) < 0.000001	-0.0443 (-0.0561, -0.0325) < 0.000001
Q4(42.53–61.90)	-0.0799 (-0.1087, -0.0512) < 0.000001	-0.0301 (-0.0615, 0.0013) 0.080447	-0.0681 (-0.0800, -0.0563) < 0.000001	-0.0734 (-0.0871, -0.0598) < 0.000001
Gynoid LM%				
Q1(38.10-57.47)	reference	reference	reference	reference
Q2(57.47–64.93)	0.0387 (0.0105, 0.0669) 0.007235	0.0263 (0.0207, 0.0319) < 0.000001	0.0270 (0.0196, 0.0344) < 0.000001	0.0292 (0.0210, 0.0373) < 0.000001
Q3(64.93–71.92)	0.0640 (0.0359, 0.0920) 0.000008	0.0456 (0.0360, 0.0551) < 0.000001	0.0494 (0.0397, 0.0592) < 0.000001	0.0548 (0.0430, 0.0665) < 0.000001
Q4(71.92–88.18)	0.0799 (0.0512, 0.1087) < 0.000001	0.0301 (-0.0013, 0.0615) 0.080447	0.0681 (0.0563, 0.0800) < 0.000001	0.0734 (0.0598, 0.0871) < 0.000001
Arm FM%				
Q1(11.20-24.09)	reference	reference	reference	reference
Q2(24.09–32.19)	-0.0200 (-0.0266, -0.0134) < 0.000001	-0.0151 (-0.0363, 0.0062) 0.165192	-0.0208 (-0.0284, -0.0132) < 0.000001	-0.0255 (-0.0341, -0.0168) < 0.000001
Q3(32.19–42.79)	-0.0620 (-0.0729, -0.0511) < 0.000001	-0.0338 (-0.0543, -0.0133) 0.001258	-0.0495 (-0.0598, -0.0392) < 0.000001	-0.0621 (-0.0743, -0.0498) < 0.000001
Q4(42.79–66.77)	-0.1399 (-0.1652, -0.1146) < 0.000001	-0.0620 (-0.0835, -0.0405) < 0.000001	-0.0809 (-0.0943, -0.0674) < 0.000001	-0.0947 (-0.1101, -0.0794) < 0.000001
Arm LM%				
Q1(31.44–53.66)	reference	reference	reference	reference
Q2(53.66–63.92)	0.0671 (0.0391, 0.0951) 0.000003	0.0203 (0.0139, 0.0267) < 0.000001	0.0228 (0.0147, 0.0310) < 0.000001	0.0233 (0.0144, 0.0322) < 0.000001
Q3(63.92–71.85)	0.1065 (0.0783, 0.1347) < 0.000001	0.0281 (0.0174, 0.0388) < 0.000001	0.0466 (0.0357, 0.0574) < 0.000001	0.0528 (0.0399, 0.0657) < 0.000001
Q4(71.85–84.68)	0.1196 (0.0904, 0.1489) < 0.000001	0.0483 (0.0243, 0.0723) 0.000080	0.0633 (0.0498, 0.0768) < 0.000001	0.0716 (0.0562, 0.0869) < 0.000001
Leg FM%				
Q1(10.80-26.68)	reference	reference	reference	reference
Q2(26.68–34.47)	-0.0218 (-0.0279, -0.0157) < 0.000001	-0.0290 (-0.0570, -0.0011) 0.041987	-0.0233 (-0.0305, -0.0161) < 0.000001	-0.0258 (-0.0340, -0.0177) < 0.000001
Q3(34.47–42.94)	-0.0580 (-0.0678, -0.0482) < 0.000001	-0.0467 (-0.0740, -0.0195) 0.000784	-0.0557 (-0.0653, -0.0461) < 0.000001	-0.0484 (-0.0598, -0.0369) < 0.000001
Q4(42.94–61.84)	-0.0927 (-0.1203, -0.0651) < 0.000001	-0.0779 (-0.1054, -0.0504) < 0.000001	-0.0852 (-0.0967, -0.0737) < 0.000001	-0.0842 (-0.0976, -0.0709) < 0.000001
Leg LM%				
Q1(35.74–54.11)	reference	reference	reference	reference
Q2(54.11–62.16)	0.0334 (0.0049, 0.0619) 0.021715	0.0266 (0.0210, 0.0323) < 0.000001	0.0267 (0.0195, 0.0340) < 0.000001	0.0286 (0.0204, 0.0369) < 0.000001
Q3(62.16–69.65)	0.0677 (0.0394, 0.0961) 0.000003	0.0387 (0.0293, 0.0480) < 0.000001	0.0546 (0.0450, 0.0642) < 0.000001	0.0497 (0.0381, 0.0613) < 0.000001
Q4(69.65–85.04)	0.0752 (0.0463, 0.1041) < 0.000001	0.0566 (0.0266, 0.0867) 0.000219	0.0702 (0.0588, 0.0817) < 0.000001	0.0583 (0.0449, 0.0717) < 0.000001
Torso FM%				
Q1(10.60-25.77)	reference	reference	reference	reference
Q2(25.77–32.07)	-0.0272 (-0.0345, -0.0200) < 0.000001	-0.0217 (-0.0312, -0.0122) 0.000007	-0.0208 (-0.0279, -0.0137) < 0.000001	-0.0340 (-0.0428, -0.0253) < 0.000001
Q3(32.07–38.58)	-0.0560 (-0.0660, -0.0460) < 0.000001	-0.0222 (-0.0317, -0.0127) 0.000005	-0.0417 (-0.0505, -0.0329) < 0.000001	-0.0474 (-0.0577, -0.0372) < 0.000001
Q4(38.58–56.27)	-0.1227 (-0.1403, -0.1052) < 0.000001	-0.0537 (-0.0653, -0.0422) < 0.000001	-0.0739 (-0.0864, -0.0615) < 0.000001	-0.0849 (-0.0984, -0.0714) < 0.000001
Torso LM%				
Q1(42.78–60.06)	reference	reference	reference	reference
Q2(60.06–66.40)	0.0582 (0.0439, 0.0724) < 0.000001	0.0273 (0.0201, 0.0344) < 0.000001	0.0278 (0.0191, 0.0365) < 0.000001	0.0311 (0.0221, 0.0401) < 0.000001
Q3(66.40-72.55)	0.0829 (0.0675, 0.0984) < 0.000001	0.0273 (0.0176, 0.0370) < 0.000001	0.0468 (0.0365, 0.0571) < 0.000001	0.0421 (0.0311, 0.0530) < 0.000001
Q4(72.55–86.90)	0.1036 (0.0858, 0.1215) < 0.000001	0.0398 (0.0282, 0.0514) < 0.000001	0.0618 (0.0494, 0.0743) < 0.000001	0.0684 (0.0549, 0.0820) < 0.000001
Total FM%				
Q1(11.69–26.29)	reference	reference	reference	reference
Q2(26.29–32.33)	-0.0237 (-0.0304, -0.0170) < 0.000001	-0.0183 (-0.0323, -0.0043) 0.010667	-0.0218 (-0.0292, -0.0144) < 0.000001	-0.0289 (-0.0375, -0.0204) < 0.000001
Q3(32.33–39.53)	-0.0710 (-0.0813, -0.0608) < 0.000001	-0.0369 (-0.0505, -0.0232) < 0.000001	-0.0521 (-0.0615, -0.0427) < 0.000001	-0.0687 (-0.0798, -0.0576) < 0.000001
Q4(39.53–55.37)	-0.1396 (-0.1619, -0.1174) < 0.000001	-0.0679 (-0.0828, -0.0530) < 0.000001	-0.0824 (-0.0950, -0.0697) < 0.000001	-0.1047 (-0.1190, -0.0905) < 0.000001
Total LM%				
Q1(42.99–57.75)	reference	reference	reference	reference
Q2(57.75–64.70)	0.0623 (0.0409, 0.0837) < 0.000001	0.0244 (0.0178, 0.0309) < 0.000001	0.0248 (0.0165, 0.0331) < 0.000001	0.0285 (0.0195, 0.0375) < 0.000001
Q3(64.70–70.50)	0.0978 (0.0758, 0.1198) < 0.000001	0.0418 (0.0320, 0.0515) < 0.000001	0.0523 (0.0421, 0.0626) < 0.000001	0.0573 (0.0454, 0.0692) < 0.000001
Q4(70.50-84.37)	0.1135 (0.0900, 0.1369) < 0.000001	0.0455 (0.0295, 0.0615) < 0.000001	0.0668 (0.0541, 0.0795) < 0.000001	0.0788 (0.0643, 0.0933) < 0.000001

## Discussion

In this study, we investigated the relationship between segmental body composition and total BMD. Our analysis unveiled that FM% and LM% in various anatomical regions exhibited a significant association with total BMD. Remarkably, this association remained robust after adjusting for relevant confounding variables and proved consistent across different gender and age groups. FM% displayed an inverse correlation with total BMD, with the most significant impact originating from FM% in both the upper and lower extremities. Conversely, our exploration uncovered a positive relationship between LM% and BMD across all anatomical regions. These findings underscored the significance of incorporating segmental body composition as a critical factor in the assessment of the variables influencing BMD.

The distribution of adipose tissue exerts a profound impact on systemic metabolism, increasing susceptibility to metabolic disorders. The pivotal determinant in this interplay lies in the diverse capability of adipose tissue to generate bioactive molecules that can impact various bodily tissues [30, 31]. Studies have demonstrated inherent genetic and developmental differences in adipocytes across distinct anatomical regions, with varying associations with BMD [32, 33]. For instance, previous research by Kuwahata et al. [34] suggested that trunk fat mass, owing to its non-weight-bearing effect, might exert a more significant impact on BMD augmentation than peripheral fat mass. Moreover, Matsuo et al. [35] found that in premenopausal women, fat distribution in the upper body had a stronger association with regional bone density than that in the lower extremities. Furthermore, studies on Korean adolescents indicated that fat mass in the torso area might enhance bone density more than in the extremities [36]. Douchi et al. [37] observed a greater impact of upper-body fat on BMD in premenopausal Japanese women compared to overall adiposity. However, it is essential to note that our findings differ from these prior investigations. These disparities may be attributed to the relatively modest sample sizes in most studies, typically involving only dozens or hundreds of subjects, as well as differences in the age, gender, and ethnicity of the cohorts under examination. Discrepancies also arise from the covariates considered in the correlation analyses within the respective models. In our study, we found that the distribution of adipose tissue across all anatomical regions, whether central or peripheral, upper body or lower body, demonstrated an inverse correlation with BMD. Surprisingly, the most pronounced impact on BMD was observed in the legs and arms, despite these regions not having the highest proportion of adipose tissue.

The adipose depots in the arms and legs primarily consist of subcutaneous fat, while the central region contains a combination of visceral and subcutaneous fat. A study [34] suggested that visceral fat might have a greater positive influence on BMD compared to subcutaneous fat, attributed to the biochemical components generated by adipocytes. On the contrary, trunk adipose tissue, due to its non-weight-bearing effect, was inherently predisposed to more effectively enhance BMD than peripheral fat. Furthermore, investigations had illuminated the adverse effects of excess visceral adipose tissue on sex hormone-binding globulin (SHBG) levels, leading to an excess of free sex hormones circulating in the body. Studies revealed a robust correlation between SHBG and fat distribution in the Android region, resulting in elevated levels of free estrogen and testosterone, which can play pivotal roles in promoting higher BMD [35, 38]. An observational study suggested that estrogen regulation might elucidate the favorable link between adipose tissue in the Gynoid region and BMD [39]. Additionally, Omentin, a specific adipose factor synthesized and secreted in visceral adipose tissue [40], had been demonstrated to play an essential role in modulating the activities of osteoblasts and osteoclasts, providing a promising avenue for improving osteoporosis and reducing fracture risk [41]. Hence, our hypothesis revolved around the idea that, despite the negative correlation between visceral fat and BMD, there may be an underlying mechanism within visceral fat that promotes bone health. This suggested that subcutaneous fat might pose a greater risk to bone health when compared to visceral fat.

Recent research had substantiated the divergent implications of adipose accumulation in the upper and lower body in relation to the susceptibility to obesity-related metabolic disorders and complications [42]. Interestingly, adipose tissue in the lower body appeared to provide protection against these health risks [43, 44]. Remarkably, our investigation unveiled a negative correlation between adipose tissue in the legs and BMD. One plausible explanation lies in the influence of sex hormones, recognized for their critical role in bone growth and maintenance [45, 46]. Studies had observed an inverse correlation between androgenic activity and lower extremity adipose tissue [47] and reduced androgenic activity in lower body adipose depots [48]. Moreover, the association between leg fat and BMD may be intricately intertwined with physical activity. Physical activity and exercise routines are steadfast defenders of musculoskeletal integrity, facilitating adipose reduction and guarding against osteoporosis [49, 50]. Lower extremity adipose tissue proportion may serve as an indicator of physical activity. An insightful study involving stroke patients shed light on the detrimental consequences of physical inactivity, emphasizing its contribution to the accumulation of adipose deposits in the lower limbs [51]. Concurrently, resistance-based exercise routines have been demonstrated to reduce lower limb adiposity [52]. Additionally, intramuscular adipose tissue in the femoral compartment may exert an adverse influence on motor function and physical activity levels [53]. Therefore, a vicious cycle between inactivity and the accumulation of adipose tissue may develop. Consequently, a lack of exercise contributes to augmented adiposity within the lower limbs and a concurrent decrease in bone mass.

Our investigation revealed a significant association between body composition across various anatomical regions and BMD, particularly focusing on the adipose parameters in the arms and legs, indicating an increased risk of BMD reduction. To mitigate this risk, we emphasized the importance of gaining lean mass, which had been substantiated to wield a significant protective effect on BMD. These findings hold significant relevance in light of the global concerns surrounding obesity and osteoporosis. Both conditions have been linked to suboptimal dietary patterns, excessive calorie intake, and insufficient physical activity [54, 55]. As such, we advocate a multifaceted approach to address these issues, including high-intensity resistance exercise combined with the supplementation of calcium and protein. A calcium-rich diet assumes a critical role in regulating energy metabolism and reducing fat accumulation during periods of excessive consumption [56]. Inadequate protein intake has been associated with declines in lean mass [57]. Maintaining optimal daily protein intake is essential for preserving lean mass and preventing bone loss [58]. Furthermore, exercise intervention emerges as a dual-faceted solution, preserving lean mass while effectively managing the loss of adipose tissue [59]. Studies have shown that exercise intensity is significantly correlated with increases in lean mass and BMD, simultaneously resulting in reductions in adipose weight [60]. Consequently, the effective management of dietary habits and regular exercise is of paramount importance in addressing the intricate interplay between body composition and BMD.

While our study benefits from substantial sample size and the use of contemporary DXA data, it is essential to acknowledge several inherent limitations. Firstly, our research adopted a cross-sectional study design, which inevitably constrains our ability to establish causal relationships. Secondly, our study data primarily comprises non-institutionalized individuals in the United States, specifically within the age range of 20 to 59 years, limiting the generalization of our findings to various age groups, races, and ethnicities. Additionally, despite our best efforts, it is plausible that the association between segmental fat distribution and BMD in American adults may still be influenced by unaccounted-for confounding variables. Lastly, our study centered on total BMD for analysis, even though it acknowledges its positive correlation with regional BMD. It is conceivable that variations in the correlation between segmental body composition and regional BMD may exist, a facet that our study did not explore.

## Conclusions

In this population-based study conducted in the United States, we discovered that an elevated proportion of adipose tissue across various anatomical regions poses a risk factor for BMD reduction. Furthermore, the protective role of lean mass in the preservation of bone health had been validated by our research. These associations showed consistency across gender and age groups. Consequently, the inclusion of segmental body composition analysis in clinical assessments, with specific attention to the fat ratio of legs and arms, should be considered highly relevant for both predictive and preventive measures against the deleterious effects of osteoporosis. However, to further strengthen and substantiate these findings, additional prospective cohort studies should be required.

## Data Availability

Publicly available datasets were analyzed in this study. This data can be found here: https://www.cdc.gov/nchs/NHANES/index.htm. The datasets generated and analyzed during the current study are available in the ZENODO repository, 10.5281/zenodo.7661039.

## Abbreviations

BMD: Bone mineral density
BMI: Body mass index
CI: Confidence intervals
DXA: Dual-energy X-ray absorptiometry
FM%: Fat mass percentage
LM%: Lean mass percentage
NHANES: National Health and Nutrition Examination Survey
SHBG: Sex hormone-binding globulin
